# Antibacterial, angiogenic, and osteogenic activities of Ca, P, Co, F, and Sr compound doped titania coatings with different Sr content

**DOI:** 10.1038/s41598-019-50496-3

**Published:** 2019-10-02

**Authors:** Jianhong Zhou, Xiaoli Wang, Lingzhou Zhao

**Affiliations:** 1 Institute of Physics & Optoelectronics Technology, Advanced Titanium Alloys and Functional Coatings Cooperative Innovation Center, Baoji University of Arts and Sciences, Baoji, 721016 China; 2 School of Marxism, Baoji University of Arts and Sciences, Baoji, 721016 China; 3 State Key Laboratory of Military Stomatology & National Clinical Research Center for Oral Diseases & Shaanxi Engineering Research Center for Dental Materials and Advanced Manufacture, School of Stomatology, The Fourth Military Medical University, Xi’an, 710032 China

**Keywords:** Implants, Orthopaedics

## Abstract

Titanium implants are often combined with microporous titania coatings simultaneously doped with various elements to enhance their antibacterial, angiogenic and osteogenic activities. To evaluate how Sr doping levels affect properties of titania coatings simultaneously doped with Ca, P, Co and F (TiCPCF coatings), we prepared coatings with Sr contents equal to 6, 11 and 18 wt% (TiCPCF-S6, TiCPCF-S11 and TiCPCF-S18, respectively) using micro-arc oxidation of titanium. Sr presence in TiCPCF coatings did not affect their phase compositions, microstructure, surface wettability, roughness, and adhesion to titanium. Antibacterial, angio- and osteo-genic activities of all the coatings were evaluated. Sr incorporation improved mesenchymal stem cell proliferation, osteogenic differentiation and implant osseointegration. TiCPCF-S11 showed the most optimum Sr content judging by its enhanced osteogenic activity. While Sr incorporation did not weaken angiogenic and antibacterial abilities of TiCPCF. Thus TiCPCF-S11 coating is a very strong candidate to be used as a next-generation bone implant material.

## Introduction

Titanium (Ti) is a popular metal used for fabrication of body implants because of its excellent mechanical performance and biocompatibility^1^. Yet, even though metallic Ti is inert in physiological conditions and environment, it shows poor antibacterial activity, and, as a result, implantation failure can occur from the implant-related infections^2^ and poor implant osseointegration^3^. At the same time, it is more and more recognized that good neovascularization provides new-forming bones with necessarily nutrition and facilitates migration of mesenchymal stem cells (MSCs) to the implant surface to form or to restore a bone or bone tissues^4^. Therefore, next-generation advanced Ti-based implant coatings should exhibit multifunctionality, such as enhanced antibacterial, osteogenic and angiogenic activities.

Incorporation of inorganic bioactive elements with slow delivering speeds provides implants with necessarily and needed enhanced antibacterial, angiogenic and osteogenic abilities. These additives should be stable and must enhance their incorporation with high efficiency even at low concentrations. Thus, lasting antibacterial, angiogenic and osteogenic effects and properties can be accomplished by adjusting doses as well as release rates of these inorganic bioactive elements^5^ incorporated into Ti-implants. Among variety of the inorganic bio-additives, fluorine (F) demonstrates excellent antibacterial properties as well as osteogenic activity^6,7^. For angiogenic activities, cobalt (Co) increases cellular stimuli response of hypoxia-inducible factors promoting angiogenesis. Co-containing mesoporous bioactive glass and TiO_2_ coatings deposited on metallic Ti demonstrated improved angiogenic properties^8,9^. Recently, strontium (Sr) attracted significant clinical attention in the field of bone formation: presence of strontium can increase osteoblast differentiation, replication and bone matrix mineralization. Additionally, Sr presence can also guide MSC transport to the growing bones^10,11^. In this work, we implemented micro-arc oxidation (MAO), which, when used to incorporate inorganic dopants into metallic Ti implant surfaces, yields rough, strongly adhering and bioactive titania (TiO_2_)-based coatings^12,13^.

Our previous works showed that incorporation of Sr, Co, F, Ca and P into TiO_2_ coating results in better osteogenic and angiogenic as well as antibacterial activities^14^, and have optimized the F6 and Co9 contents in the coatings. However, when Sr contents are too high, deterioration of these properties (e.g., inhibition of proliferation, osteogenic differentiation of cells, etc.) might occur^15,16^. Therefore, it is essential to optimize Sr doping levels in these coatings and to study how Sr content affects release of other bioactive elements as well as their functional interactions. In this work, microporous TiO_2_ coatings doped with the same amounts of Ca, P, Co and F (TiCPCF coatings) but with different amounts of Sr contents (∼6, ∼11 and ∼18 wt%) (TiCPCF-S6, TiCPCF-S11 and TiCPCF-S18, respectively) were fabricated directly on metallic Ti substrates (wires and disks) using MAO. Bone marrow MSCs from New Zeland rabbits, *Staphylococcus aureus* (*S*. *aureus*) and *Escherichia coli* (*E*. *coli*) bacteria were implemented to analyze antibacterial, angio- and osteo-genic properties of these coatings both *in vitro* and *in vivo*.

## Results

### Coating characterization

**Figure 1:**
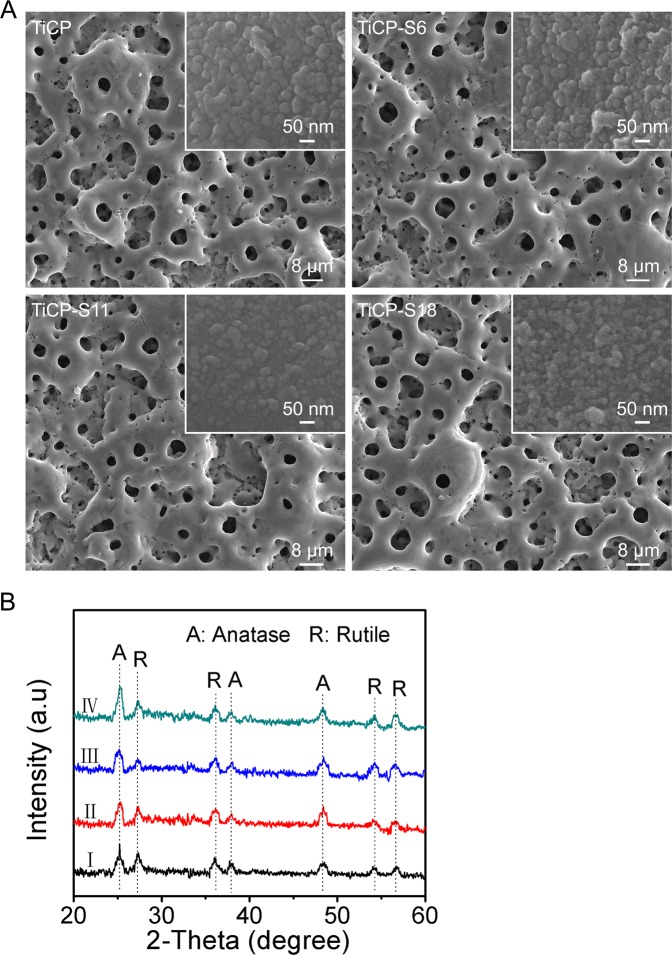
(**A**) SEM images and (**B**) TF-XRD patterns of TiCPCF, TiCPCF-S6, TiCPCF-S11 and TiCPCF-S18 coatings.

TiCPCF, TiCPCF-S6, TiCPCF-S11 and TiCPCF-S18 coatings demonstrated standard microporous MAO morphology with 3–4 μm pores distributed homogeneously (Fig. 1A). High magnification analysis revealed ~30–60 nm nanograins (insert on top of Fig. 1A). XPS demonstrated Sr presence in TiCPCF-S6, TiCPCF-S11 and TiCPCF-S18, confirming its successful incorporation (Table S3). All coatings contained Ti, O, F, P, Ca and Co. Sr content differed from coating to coating but contents of all other elements were at the same level. Sr content in coatings was adjusted by using different strontium acetate concentration in MAO electrolytes: the more Sr was in the electrolyte, the higher its content was in the coating. All coatings consisted of anatase and rutile phases (Fig. 1B). Incorporation of Ca, P, Sr, Co and F slightly change rutile/anatase ratio in the coatings. No detectable XRD peaks corresponding to the phases containing Ca, F, P, Co and/or Sr were detected.

**Figure 2:**
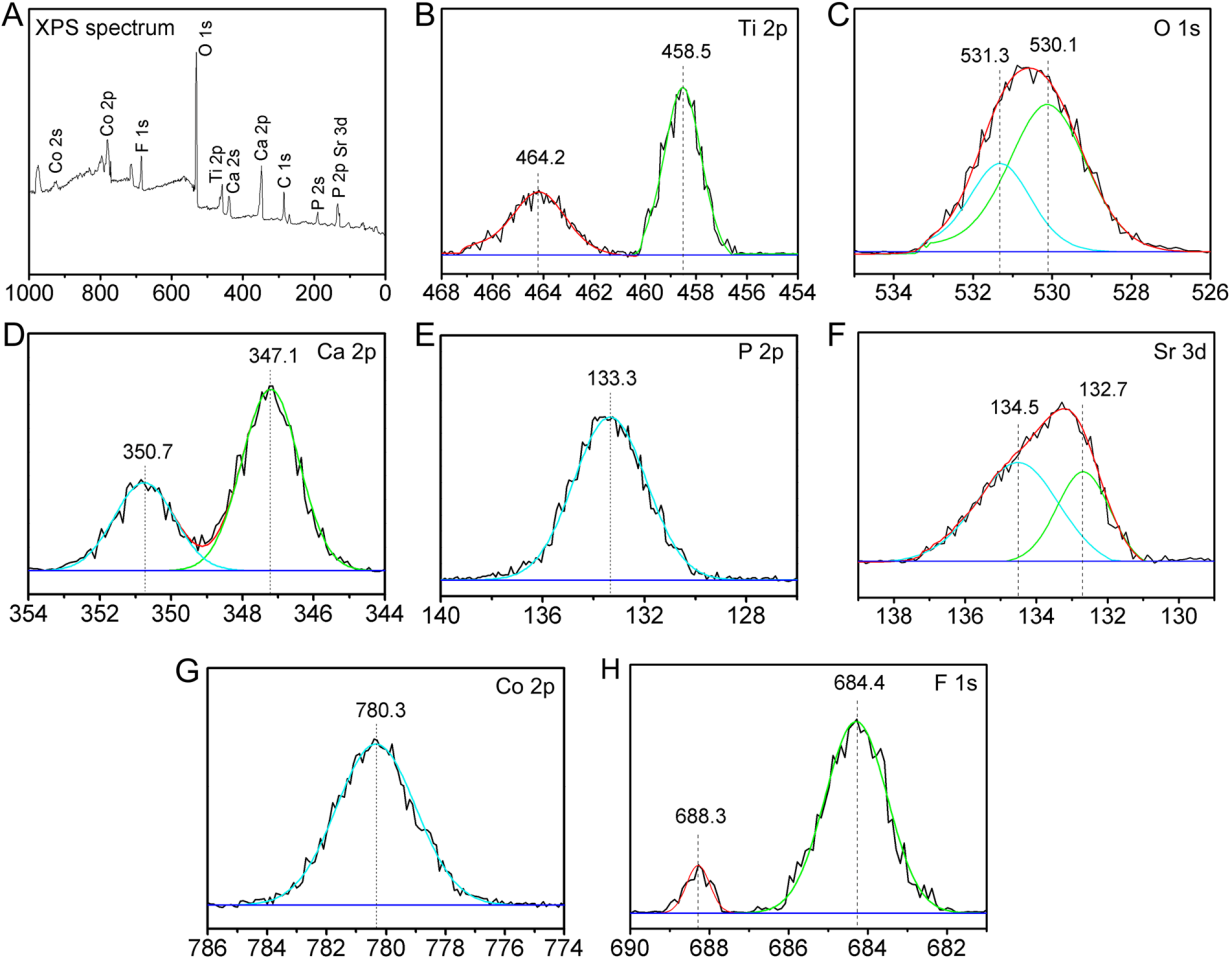
Full (**A**) and individual elemental XPS spectra of Ti2p (**B**), O1s (**C**), Ca2p (**D**), P2p (**E**), Sr3d (**F**), Co2p (**G**) and F1s (**H**) obtained from the surface of TiCPCF-S11 coating.

XPS full spectrum of TiCPCF-S11 confirmed simultaneous presence of Co, Sr, F, Ca and P in the coating (Fig. 2A). High-resolution Ti2p spectrum demonstrated standard binding energy peaks of TiO_2_^17^ (Fig. 2B). O1s spectrum was split into two constituent Gaussian peaks (Fig. 2C). Peak at 530.1 eV belongs to O1s in TiO_2_^18^. Peak at 531.3 eV was attributed to O1s in phosphate groups of CaHPO_4_ and/or Ca_3_(PO_4_)_2_^19^. Ca2p peaks were detected at 347.1 and 350.7 eV (Fig. 2D). P2p peak was at 133.3 eV (Fig. 2E). Thus, in the coatings, Ca and P co-existed in calcium phosphate-based phases (e.g. amorphous calcium phosphate and α-tricalcium phosphate)^20^. Doublet XPS Sr3d peaks were observed at ~132.7 and 134.5 eV, which are typical peak positions for SrTiO_3_^21^ (Fig. 2F). Co2p peak at 780.3 eV was assigned to Co2p from CoTiO_3_^22^ (Fig. 2G). F1s peaks were detected at 684.4 and 688.3 eV. Thus, F in the coating was in the Ti-F form and in the TiO_2_ lattice^23,24^ (Fig. 2H). These results are similar to our previous work^14^, indicating that different levels of incorporated Sr did not affect chemical states and properties of other doping elements.

Data on wettability and roughness of all the coatings as well as on metallic Ti are shown in Table S4. Average roughness (Ra), root-mean-square roughness (RMS),and selection of 10-point height of irregularity roughness (Rz) demonstrated similar sub-micron roughness characteristics for all coatings. All coatings demonstrated similar values of water contact angles and BET surface areas. Thus, presence of different levels of Sr in the coatings did not significantly affect the wettability, roughness and BET surface areas of all the coatings.

### Adhesion strength and ion release of all the coatings

**Figure 3:**
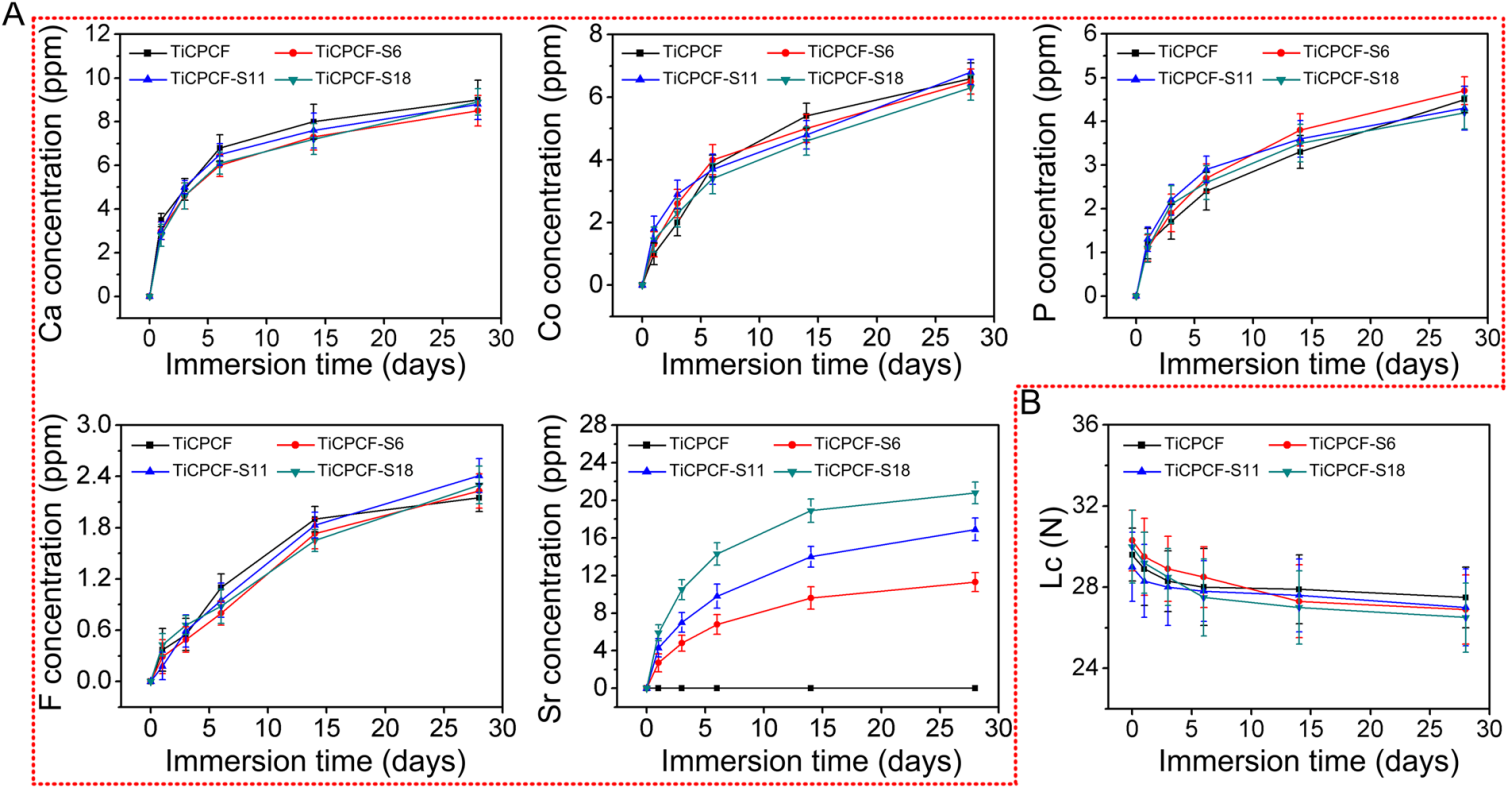
(**A**) Cumulative levels of calcium, cobalt, phosphorus, fluorine and strontium leached from the coatings immersed into PS solutions. (**B**) Adhesion before and after coating immersion in PBS.

*A*dhesion strength and ion release of all the coatings after being immersed in PS solution are shown in Fig. 3. All elements (F, Ca, P, Co, and Sr) were found in the leachate solutions obtained from all coatings. Their concentrations increased with prolonged immersion time, which is indicative of their continuous release. Sr demonstrated a controlled release profile; and its release level positively correlated with the initial Sr amount in the coating, which increased in the following order: TiCPCF-S18 > TiCPCF-S11 > TiCPCF-S6 (Fig. 3A). Presence of Sr in the coating did not noticeably affect release profiles of P, Ca, F and Co (Fig. 3A).

Coating adhesion to the metallic Ti substrate after prolonged exposure to PS solution is shown in Fig. 3B. Lc values of all coatings before their exposure to PS were ~30.6 N on average. This value corresponds to strong and stable bonding. After being soaked in PS solution for up to 4 weeks, adhesion of all coatings remained strong with only some slight decrease in Lc values. Thus, all coatings tested in this work possessed long-term stability and adhesion to metallic Ti even after prolonged immersion in simulated physiological conditions.

### Cytotoxicity, protein adsorption, proliferation, cell adhesion and morphology characteristics and properties of the coatings

**Figure 4:**
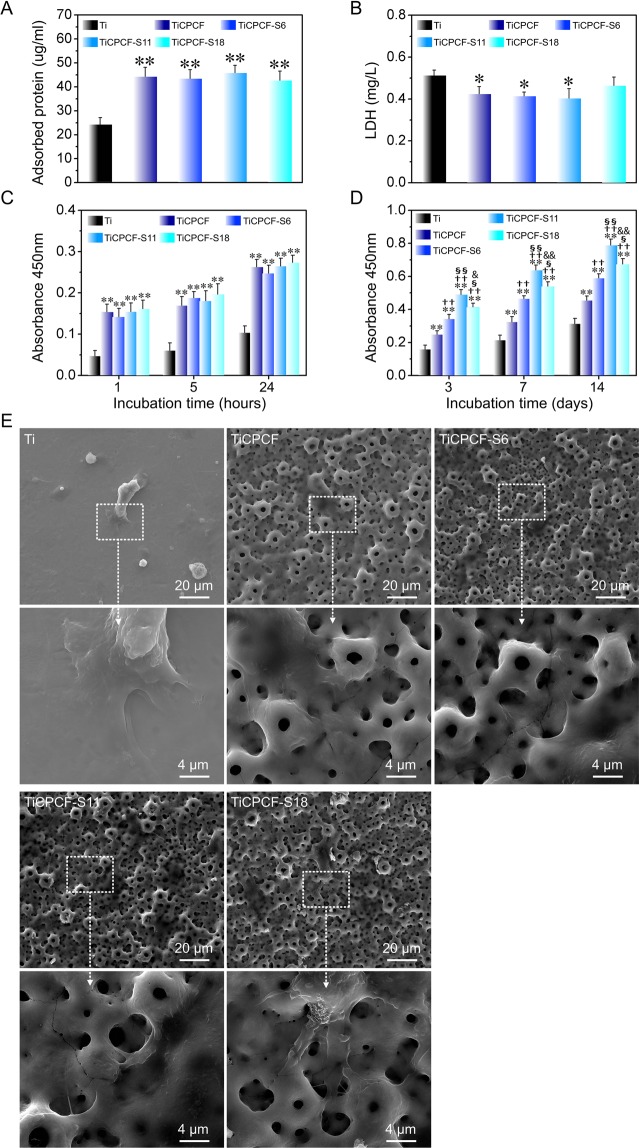
(**A**) Proteins adsorbed by the substrates during immersing in a-MEM solution with 10% FBS for 24 hours. (**B**) Amounts of LDH released by MSCs 3 days after the incubation. MSC adhesion (**C**) and proliferation (**D**) obtained using CCK-8 assay kit for samples cultured for 1, 5 and 24 hours (**C**) and for 3–14 days (**D**), respectively. (**E**) SEM micrographs of MSCs cultured for 3 days. All values are averages calculated from five independent measurements. Errors are standard deviations. *p < 0.05 and **p < 0.01 com*p*ared to metallic Ti substrate; ^††^*p < *0.01 compared to TiCPCF coating; ^§^*p < *0.05 and ^§§^*p < *0.01 compared to TiCPCF-S6 coating; ^&^*p < *0.05 and ^&&^*p < *0.01 compared to TiCPCF-S11 coating.

Protein adsorption to the implant is a typical biological response from the surrounding cells and tissues^25^. Data on protein adsorption after immersing in α-MEM with 10% FBS for 24 h is shown in Fig. 4A. TiCPCF, TiCPCF-S6, TiCPCF-S11 and TiCPCF-S18 adsorbed more proteins than unmodified metallic Ti because of their rougher and larger surfaces favorable for protein attachment. All coatings demonstrated similar amounts of adsorbed proteins. Thus, incorporation of Ca, F, P, Co, and Sr did not affect protein adsorbing properties of the coatings.

All coatings exhibited no cytotoxicity, which was judged by the LDH released by the cultured MSCs, comparing to metallic Ti. Thus, incorporation of P, Ca, Sr and F at the levels tested in this work and their release rates, levels and mechanisms were safe for the conditions tested in this work. LDH levels induced by metallic Ti were slightly higher comparing to those of the coatings. Thus, coatings demonstrated enhanced cytocompatibility, which was also confirmed by *in vitro* proliferation and cell adhesion test results.

Proliferation of cells and differentiation strongly depend on initial cell adhesion^26^. More initial adherent cells were observed for coatings than for metallic Ti substrate most likely because of enhanced protein adsorption and larger surface area of the coatings (Fig. 4C). Number of initial adherent cells was somewhat similar for all the coatings. Thus, incorporation of different levels of Sr did not noticeably affect amounts of initial adherent cells. MSCs proliferated well on all substrates. Their amounts on different substrates after 3–14 days of incubation were in the following order: TiCPCF-S11 > TiCPCF-S18 > TiCPCF-S6 > TiCPCF > Ti.

Cell functions of biomaterials strongly correlate with their shapes^27^. FE-SEM analysis of how MSCs adhered and spread after being cultured for 3 days showed that on pure metallic Ti, MSCs expanded poorly in a spindle-like manner, which is typical for undifferentiated inactive cells (Fig. 4E). MSC attachments to our microporous coatings were much better: MSCs spread out extensively, simultaneously covering and attaching to the microporous structure (Fig. 4E). Presence of Sr in the coatings did not affect MSC spreading.

### *In vitro* osteogenic activity

**Figure 5:**
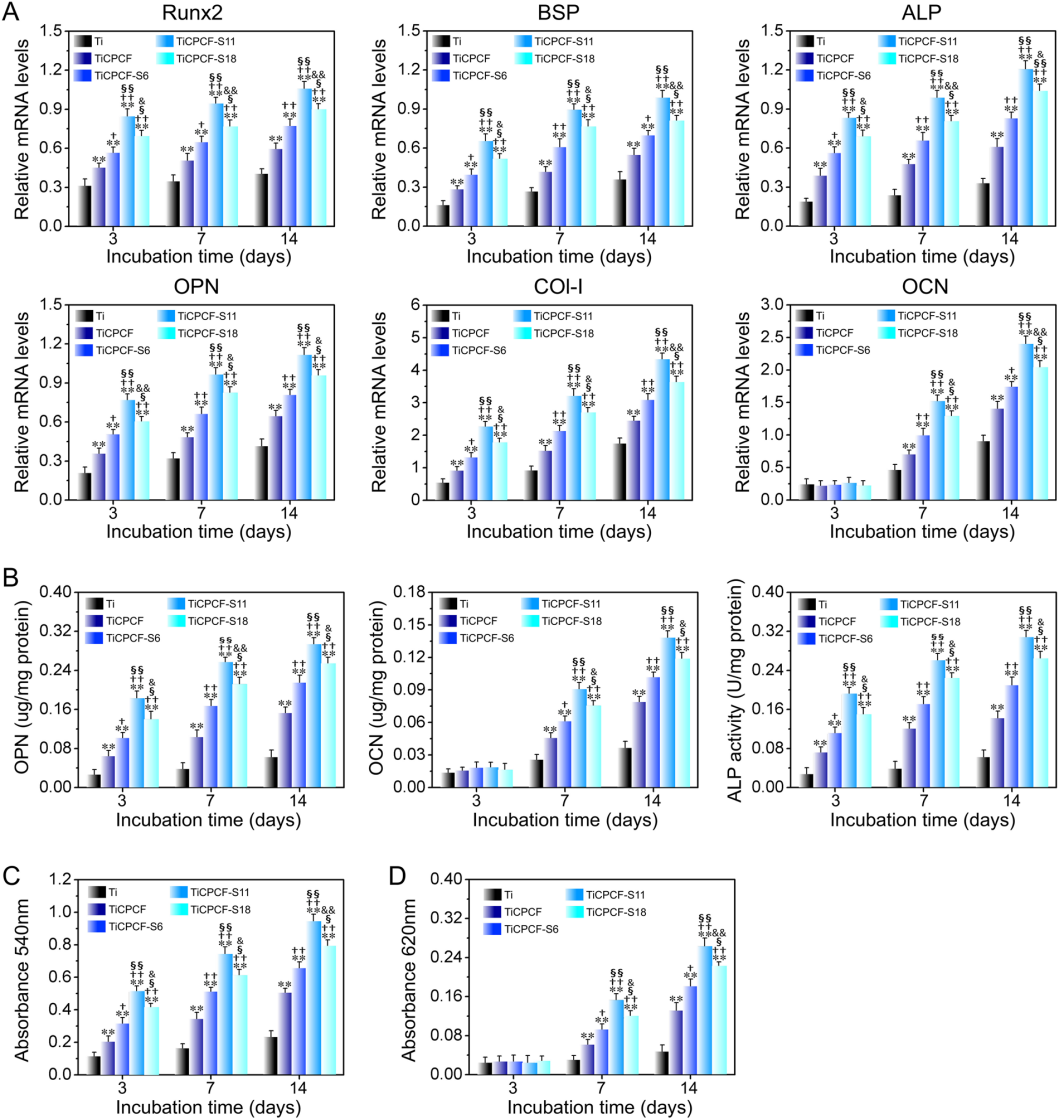
(**A**) Osteogenesis-related gene expression of BSP, Runx2, OPN, ALP, Col-I and OCN. (**B**) Intracellular protein expression of OPN, ALP, and OCN, (**C**) collagen secretion and (**D**) ECM mineralization of MSCs incubated for 3–14 days. All values are averages calculated from four independent measurements. Errors are standard deviations. **p < 0.01 compared to metallic Ti substrate; ^†^*p < *0.05 and ^††^*p < *0.01 compared to TiCPCF coating; ^§^*p < *0.05 and ^§§^*p < *0.01 compared to TiCPCF-S6 coating; ^&^*p < *0.05 and ^&&^*p < *0.01 compared to TiCPCF-S11 coating.

The osteogenesis-related genes expressions are shown in Fig. 5A. Intracellular OCN and OPN protein contents as well as ALP activity are shown in Fig. 5B. Data on MSC extracellular matrix (ECM) mineralization and collagen secretion after being cultured for 3–14 days are shown in Fig. 5C,D. Surrogate markers for cell osteogenic differentiation on different substrates were in the following order: TiCPCF-S11 > TiCPCF-S18 > TiCPCF-S6 > TiCPCF > Ti. Thus, all these coatings were able to stimulate MSC osteogenic-differentiation, which correlated with Sr content in the coating. The optimum Sr content in the TiO_2_-based doped coating was 11% judging by the best osteogenic activity of TiCPCF-S11.

### *In vitro* antibacterial activity

**Figure 6:**
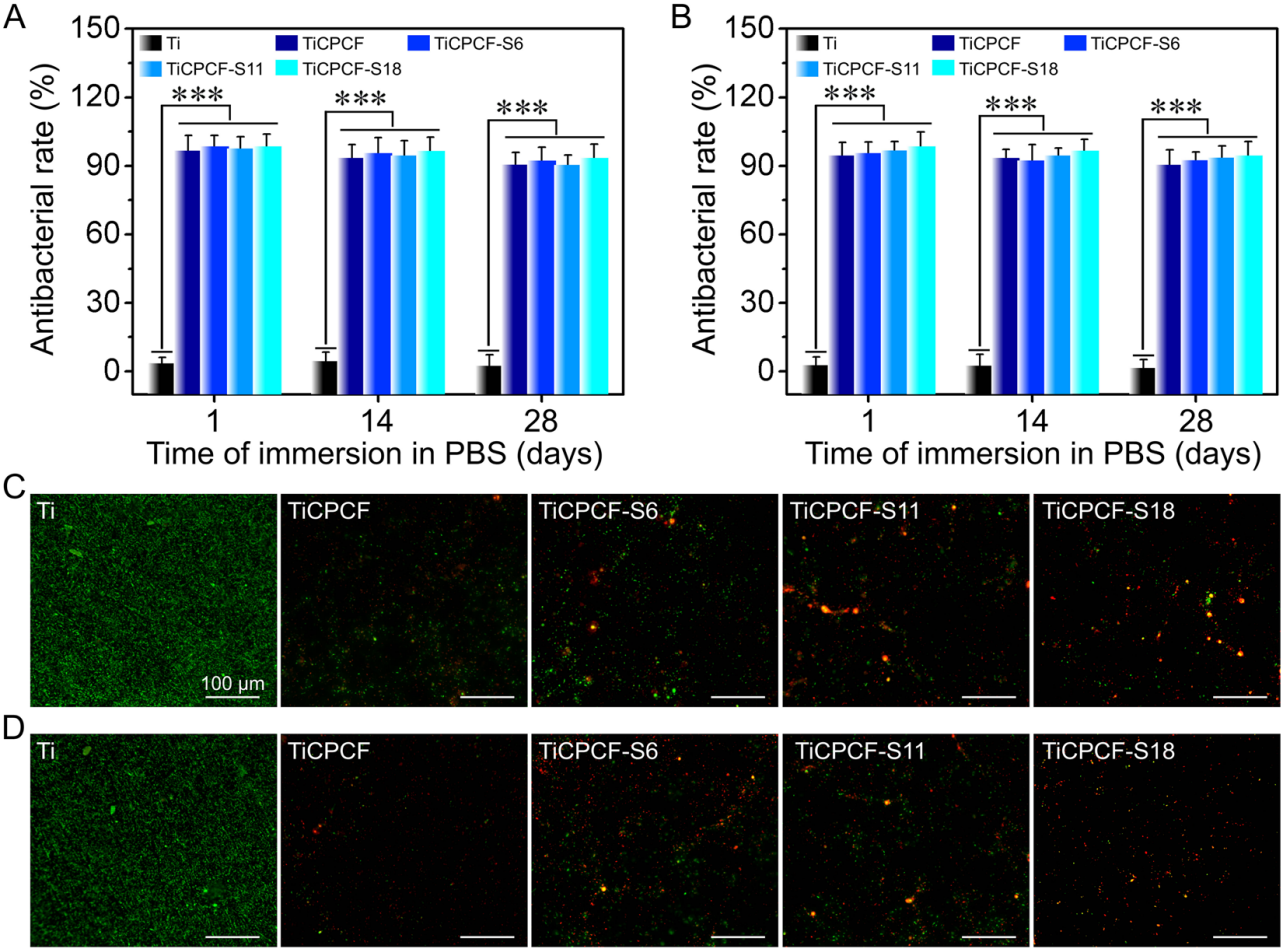
Antibacterial rates observed after substrates were immersed PBS for 1–28 days against *E*. *coli* (**A**) and *S*. *aureus* (**B**). Fluorescence images of adhered *E*. *coli* (**C**) and *S*. *aureus* (**D**) after substrates were soaked in PBS for 28 days. Died and alive bacteria are red and green in color, respectively. All values are averages calculated from four independent measurements. Errors are standard deviations ***p < 0.001 compared to metallic Ti substrate.

Data of antibacterial activity using *E*. *coli* and *S*. *aureus* indicates similar bacterial removal rates for all microporous coatings studied in this work (up to 95%, Fig. 6A,B). Even after 28-day exposure to PBS, 92% antibacterial rates were still observed for all the coatings indicating their long-term stability and antibacterial activity under physiological and biological conditions. Fluorescent staining of bacteria cultured on the substrates, which were preliminary immersed in PBS for 28 days, demonstrated significant amount of viable bacteria (shown in green color) and no died ones (which would be shown with red color) on metallic Ti substrate (Fig. 6c). At the same time, barely any viable bacteria were observed for TiO_2_-based doped microporous coatings (Fig. 6D).

### Angiogenic activity

**Figure 7:**
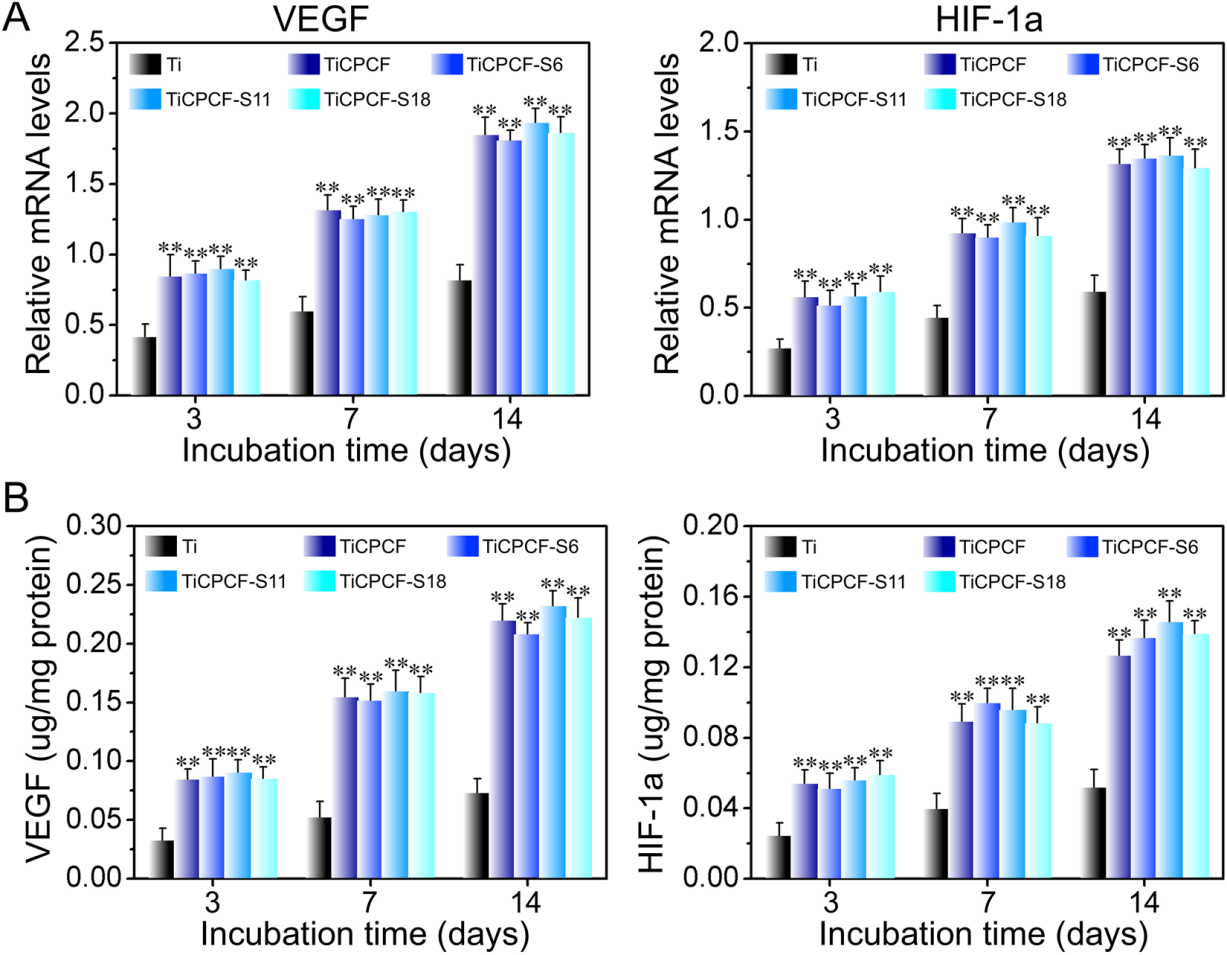
Gene expression (**A**) and intracellular protein products (**B**) of VEGF and HIF-1a in MSCs incubated on substrates for 3–14 days. Values of gene expression were normalized relative to GAPDH. All values are averages calculated from four independent measurements. Errors are standard deviations. **p < 0.01 compared to metallic Ti substrate.

Analysis of protein products and gene expression of most important angiogenic factors (HIF-1a and VEGF) in cultured MSCs showed enhanced but similar levels for both parameters for all microporous coatings. Thus, Sr presence did not affect angiogenic activity (Fig. 7).

### *In vivo* osseointegration ability

**Figure 8:**
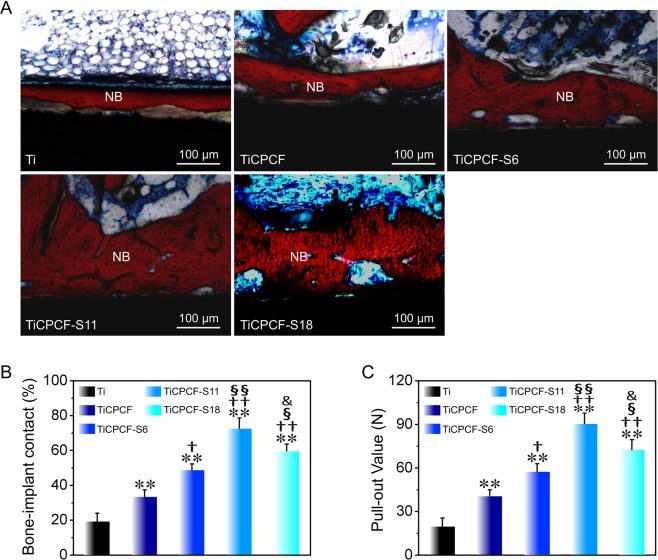
(**A**) Histological analysis of the bone/implant interface after implantation into the rabbits for 8 weeks. Red-stained tissues represent newly formed bones. Bone-to-implant contact (**B**) and pull-out force (**C**) of the metallic Ti wires with and without coatings 8 weeks after implantation. All values are averages calculated from four independent measurements. Errors are standard deviations. **p < 0.01 compared to metallic Ti substrate; ^†^*p* < 0.05, ^††^*p* < 0.01 and ^†††^*p* < 0.001 compared to TiCPCF coating; ^§^*p* < 0.05, ^§§^*p* < 0.01 compared to TiCPCF-S6 coating; ^&^*p* < 0.05 compared to TiCPCF-S11 coating.

Data on *in vivo* osteogenesis of metallic Ti wires (obtained from histological stained images) with and without coatings implanted for 8 weeks demonstrated more new bone formation for coated Ti wires (Fig. 8A). Overall amount of new bone formation was in the following order: TiCPCF-S11 > TiCPCF-S18 > TiCPCF-S6 > TiCPCF > Ti. Freshly formed bone was isolated from Ti surface by a thin layer of unmineralized fibrous tissue. At the same time, bones formed on Ti wires coated with the microporous coatings directly bonded to the coating surface without formation of an intermediate fibrous layer. Figure 8B,C show bone-to-implant contact rate and a pull-out force of the metallic Ti implant osseointegration, respectively, revealing that the general trend is TiCPCF-S11 > TiCPCF-S18 > TiCPCF-S6 > TiCPCF > Ti. Thus, all the *in vivo* data indicate that presence of Sr provided implants with better osseointegration ability comparing to Sr-free coatings and bare metallic Ti substrates. Sr doping dose of TiCPCF-S11 coating (of ~11 wt%) is the optimal level to achieve best osseointegration ability of Ti implants.

## Discussion

Our previous study found that co-incorporation of Sr, Co and F into TiO_2_ coatings already containing Ca and P can lead to better osteogenic, angiogenic, and antibacterial activities^14^ of the resulting coatings. Some studies demonstrated proliferation and osteogenic cells differentiation inhibition by high levels of Sr^15,16^. Thus, our work intended to optimize Sr incorporation dose into TiO_2_ coating already co-doped with fixed contents of Ca, P, Co and F.

Biomaterial surface properties (e,g, energy, roughness, wettability, overall chemistry and texture) affect overall performance of the coating^26^. Influence of Sr can be easily assessed if all other factors and properties (e,g, micro-porosity, surface roughness, BET surface areas and composition) are similar.

Developed in this work coatings containing different amounts of Sr (TiCPCF-S6, TiCPCF-S11 and TiCPCF-S18) improved MSCs proliferation and osteogenic differentiation, which agrees with previous literature reports^9,10,13^. We also demonstrated that MSC proliferation and osteogenic differentiation can be optimized by introducing specific Sr content (which also agrees with literature data^15,16^): proliferation and osteogenic differentiation of MSCs increased as Sr dose increased from 6 to 11%, but then decreases as Sr dose increased to 18% probably because of the saturation of the beneficial coatings properties with strontium. Similar correlation with Sr content was demonstrated for osteoblasts: small amounts of Sr stimulated their proliferation and differentiation^10,11,14^ whereas large amount inhibited the cell functions^15,16^. Our previous studies showed that simultaneous incorporation of Co and F can enhance angiogenic and antibacterial abilities of microporous coating^14^. Results obtained in this study demonstrated that further incorporation of Sr into TiCPCF coatings at contents above 18% did not affect their angiogenic and antibacterial abilities.

*In vivo* osseointegration capability was assessed by implanting coatings in live rabbits. Improvements in new bone growth and BIC ratio as well as biomechanical strength of bone-implant integration follow the order (from best to worst): TiCPCF-S11 > TiCPCF-S18 > TiCPCF-S6 > TiCPCF > metallic Ti. Incorporation of Sr induces better osseointegration than TiCPCF coating without Sr or just pure metallic Ti substrate. TiCPCF-S11 with the optimal Sr doping dose (of ~1 1 wt%) showed the best osseointegration ability. These *in vivo* osseointegration results agree with the *in vitro* results obtained from MSC proliferation and osteogenic differentiation tests.

Microporous TiO_2_ coatings co-doped with fixed Ca, P, Co and F amounts but with different Sr levels (∼6, ∼11 and ∼18 wt%) were successfully applied to metallic Ti implant using simple MAO procedure. Microstructure, surface roughness, anatase/rutile ratio as well as wettability of the TiO_2_ coatings simultaneously doped with Ca, F, Co and P were not noticeably affected by additional inclusions of Sr. All coatings demonstrated strong adhesion to Ti substrate in biological and physiological environments. Sr incorporation improved proliferation and osteogenic differentiation of MSCs as well as implant osseointegration, all of which were confirmed by both *in vitro* and *in vivo* results. TiCPCF-S11 coating with 11% of Sr showed the best osteogenic activity. Angiogenic and antibacterial abilities of the coatings were not weakened by Sr presence. We strongly believe that simultaneous incorporation of Ca, P, Co, F and Sr into microporous TiO_2_ coatings with optimum Sr content (such as TiCPCF-S11 coating developed in our study) has a very strong potential for development of practical multifunctional bone implants with advanced osteo and angio-genic as well as antibacterial abilities.

## Methods

### Micro-oxidation treatment of metallic Ti

Metallic Ti Kirschner wires (2 mm in diameter and 10 mm long) and metallic Ti disks (2 mm thick and 15 mm in diameter) were used for *in vivo* and *in vitro* experiments, respectively. Both metallic Ti wires and disks were micro-arc oxidized (MAOed) using pulse power supply in various aqueous electrolytes (all of which are listed in Table S1). Experimental parameters were 400 V/−100V pulse voltage, 100 Hz pulse frequency and 26% duty ratio applied for 5 min. The MAOed samples were ultrasonicated in alcohol, rinsed with distilled water and allowed to dry at ambient conditions.

### Sample characterization

Phase compositions were obtained using X-ray diffraction (XRD) implementing X’Pert PRO (Netherlands). Chemical compositions of all the coatings on metallic Ti were obtained by X-ray photoelectron spectroscopy (XPS) using Axis Ultra (UK). Field-emission scanning electron microscopy (FE-SEM) performed using JEOL JSM-6700F (Japan) assisted in morphology analysis of both coatings and biological samples. Surface contact-angles measured by DSA30 KRUSS (Germany) instrument, atomic force microscopy (AFM) performed using SPM-9500J3 (Japan) as well as nitrogen adsorpton-desorption isotherms were collected using ASAP2020 (America) were implemented to test coating hydrophilicity, roughness and BET surface areas, respectively.

### Assessment of ion extractions and coating adhesion

MAOed metallic Ti disks were soaked in 10 ml of 0.9 wt% NaCl physiological saline (PS) solution at 37 °C for 0, 1, 3, 6, 14, and 28 days. To measure concentrations of Sr, Ca, P, F and Co in the leachate, samples were collected at different periods and then were analyzed by inductively coupled plasma-mass spectrometry (ICP-MS) using Nu Instruments (Wrexham, UK) equipment and an F-ion electrode (9409SC, Orion Research, UK) attached to the Ion Analyzer 901 (Orion Research, UK). During F analysis, ionic strength of the sample solutions were adjusted using TISAB with CDTA buffer solutions. Calibration curve was obtained using five standard 0.1–20 ppm F solutions (purchased from Orion Research, UK). Five samples were tested for each coating.

Adhesive strength was assessed on coatings immersed in the PS solution by performing scratch tests using an auto scratch tester. Critical load value (Lc) represents failure to initiate. It was obtained by plotting load as function of the acoustic output. Final Lc value used for coating comparison was an average of five measurements.

### Protein adsorption

Incubation medium was α-Modified Eagle Medium (α-MEM) with 10% of fetal bovine serum (purchased from FBS, Life Technologies, USA). Incubation was performed on MAOed Ti disks for 24 h at 37 °C, after which the proteins were removed by 1% sodium dodecyl sulfate (Solarbio). Protein adsorption was measured by NanoDrop 2000C (Thermo Scientific, USA) at 280 nm. Final data represents average values calculated based on five measurements.

### Evaluation of antibacterial activity

Bacterial counting method using *S*. *aureus* (ATCC6538) and *E*. *coli* (ATCC10536) as gram-positive and -negative bacteria, respectively, was implemented to assess antimicrobial effects. For this purpose, the samples were soaked in 15 ml of phosphate buffer saline (PBS) solution at 37.5 °C for 1, 14, and 28 days. Fresh PBS batch was used every day. The resulting coatings were ultrasonicated, sterilized and used for antimicrobial assay (see details in Supplemental materials).

### MSCs harvesting and culture

According to the ISO 10993-2:1992 animal welfare requirements and approved by the Institutional Animal Care and Use Committee (IACUC) of Xi’an Jiaotong University. MSCs were obtained from 1-week-old New Zealand rabbits (see our previous work for details^28^). Bone marrow was removed from rabbit tibias and femora. Mononucleated cells were centrifuged and cultured at 37 °C in humid environment with 5% CO_2_ in beakers containing 20 ml of α-MEM, 10% of FBS and 1% of antibiotics. Adhered cells were collected, and non-adhered cells were discarded. All tests were conducted with cells within passage 3. 1 ml of suspension with ~2 × 10^4^ MSCs was placed on the substrates for seeding experiments.

### Morphology, cytotoxicity, proliferation and cell adhesion tests

Level of lactate dehydrogenase (LDH) activity discharged by MSCs was used to judge coating cytotoxicity. After 3 days of MSC incubation, culture media were collected and centrifuged. LDH contents in the mother solutions were measured spectrophotometrically following manufacturer instructions (Sigma, USA).

MSC proliferation and adhesion were obtained using cell counting kit CCK-8 after MSC was cultured for 1, 5 and 24 hours as well as for 3, 7 and 14 days.

For morphology analysis using FE-SEM, MSCs were first incubated for 3 days, then rinsed by PBS, then preserved by addition of 3% of glutaraldehyde and finally dried in ethanol-based freeze-drying.

### Real-time quantitative PCR assay

Expression of osteogenesis-related genes (runt-related transcription factor 2 (Runx2), osteocalcin (OCN), alkaline phosphatase (ALP), bone sialoprotein (BSP), type 1 collagen (Col-I)) and osteopontin (OPN) as well as angiogenic factors (hypoxia-inducible 1a (HIF-1a) and vascular endothelial growth (VEGF)) performed using MSCs cultured for 3 and 14 days were assessed by real-time *quantitative PCR assay*. For this purpose, 1 μg of isolated RNA from MSCs obtained by TRIzol reagent (purchased from Life Technologies, USA) was reversely transcribed onto complementary DNA using PrimeScrip RT reagent kit (purchased from TaKaRa, Japan). Expression of the genes was obtained using real-time *quantitative PCR assay* detection system (Bio-Rad iQ5 Multicolor). SYBRPremix ExTaqII (TaKaRa, Japan) coupled with iQ5 Optical System (Bio-Rad, USA) was implemented for data analysis. Expression level normalization of the target genes was performed using glyceraldehyde-3-phosphate dehydrogenase (GAPDH) gene. Primers for the target genes are shown in Table S2.

### Intracellular protein contents and ALP activity

After being cultured, cell-seeded samples were rinsed with PBS, lysed through standard five freezing and thawing cycles in 0.1 vol% of Triton X-100 (purchased from Life Technologies, USA), then agitated for 15 min. Intracellular protein (OPN, OCN, VEGF, and HIF-1a) contents and ALP activities in the cell lysates were assessed using ELISA kits (purchased from Bluegene, China). All obtained values were normalized relative to the total intracellular protein content.

### Mineralization of ECM and collagen secretion

ECM mineralization and collagen secretion of MSCs cultured for 3–14 days were assessed by staining cell-seeded samples with 0.1% Alizarin Red and 40 mM Sirius Red with pH = 4.2 (both purchased from Sigma, USA), respectively. After washing by 0.1 M acetic acid and then by distilled water, both sample stains were dissolved in 0.2 M 1:1 NaOH/methanol mixture and in 10% cetylpyridinum chloride solution (purchased from Acros Organics). The resulting solutions were used for optical density measurements at 620 and 540 nm.

### *In vivo* osteogenic activity

According to the ISO 10993-2:1992 animal welfare requirements and approved by the Institutional Animal Care and Use Committee (IACUC) of Xi’an Jiaotong University. Metallic Ti cylinders (2.5 mm in diameters and 10 mm high) with different coatings were implanted into femoral shafts of twenty-four 3-month-old mature New Zealand male rabbits, each 2–3 kg, based on the procedure from our previous work^28^. Each coating was deposited on different Ti cylinders, and then each coating was tested in five different rabbits. Eight weeks after implantation, animals were anesthetized, and the implants with attached tissues were removed to be further analyzed. Amount of bone-to-implant contacts (BIC) was obtained from 3–4 sections taken from each endosseous implant.

Biomechanical pull-out tests helped to determine strength of bone-implant integration. Femora with implants, embedded in PMMA, were collected 8 weeks after the healing. Top of each implant was positioned horizontally. Each implant was removed using Shimadzu AGS-10kNG (Japan) test machine by pulling it strictly vertically at 1 mm/min cross-head speed. Maximum pull-out force was calculated from the load-displacement curve.

### Statistical analysis

Each piece of data was obtained using four independent experiments. Error of each value was calculated as a standard deviation (SD) from the average. All data was analyzed using SPSS 14.0 software (USA). Significance level was evaluated first using one-way ANOVA and then by Student-Newman-Keuls *post hoc* tests. *p* < 0.05 and 0.01 were regarded as significant and highly significant values, respectively.

## Supplementary information

Supplementary Information
