# Supplementary material for: Antibacterial, angiogenic, and osteogenic activities of Ca, P, Co, F, and Sr compound doped titania coatings with different Sr content
Source: Sci Rep. 2019 Oct 2;9:14203. doi: 10.1038/s41598-019-50496-3 (PMC6775141; doi:10.1038/s41598-019-50496-3)
Supplement: Supplementary file 1 — Supplementary Information [file 41598_2019_50496_MOESM1_ESM.pdf]

Supplementary Information

Antibacterial, angiogenic, and osteogenic activities of Ca, P, Co, F, and Sr compound doped titania coatings with different Sr content

Jianhong Zhou[1*], Xiaoli Wang[2], Lingzhou Zhao [3]

[1] Institute of Physics & Optoelectronics Technology, Advanced Titanium Alloys and Functional Coatings Cooperative Innovation Center, Baoji University of Arts and Sciences, Baoji, 721016, China

[2] School of Marxism, Baoji University of Arts and Sciences, Baoji, 721016, China

[3] State Key Laboratory of Military Stomatology & National Clinical Research Center for Oral Diseases & Shaanxi Engineering Research Center for Dental Materials and Advanced Manufacture, School of Stomatology, The Fourth Military Medical University, Xi'an, 710032, China

**Corresponding author at: Institute of Physics & Optoelectronics Technology, Advanced Titanium Alloys and Functional Coatings Cooperative Innovation Center, Baoji University of Arts and Sciences, Baoji, 721016, China*

E-mail addresses: shanxixian198001@163.com (Jianhong Zhou)

Methods

Antibacterial activity evaluation

The antimicrobial effect of the coatings as well as Ti was evaluated by the bacterial counting method with *E. coli* (ATCC10536) and *S. aureus* (ATCC6538) as the gram-negative and the gram-positive representative, respectively. The coatings were immersed in 10 ml phosphate buffer saline (PBS) at 37°C for 1, 14, and 28 days with the PBS refreshed every day. The coatings were then ultrasonically cleaned, sterilized and employed in the antimicrobial assay. The bacteria were cultured in the beef extractpeptone (BEP, HopeBio, China) medium under agitation for 18 h at 37 °C. After dilution with BEP to a concentration of 1.0 × 105 CFU/ml, 1mL of the bacteria suspension was introduced onto each coating surface. The coatings with the bacteria suspension were incubated at 37 °C for 12h at a relative humidity of >90% in darkness. At the end of the incubation period, each coating was rinsed in phosphate buffer solution (PBS) and ultrasonically agitated to detach the bacteria from the sample. The viable bacteria in PBS were quantified by standard serial dilution and platecounting. The antibacterial activities at days 1, 14, and 28 of immersion were calculated using the following formula: R = (B - A)/B × 100%, where R is the antibacterial rate, and B and A are the mean numbers of viable bacteria (CFU) on the Ti control and the MNR samples, respectively.

Moreover, the antibacterial activities of the coantings at day 28 of immersion in PBS were vividly assayed by fluorescent staining. After incubation with the bacteria suspension for 12 h, each coating was rinsed with PBS to remove the nonadherent

bacteria. The adherent bacteria on each sample were stained with an acridine orange (AO)/propidium iodide (PI) mixture for 10 min and observed by fluorescence microscopy (DMI6000B Inverted Microscope, Leica, Germany).

Table S1. The corresponding MAO electrolyte compositions.

Coatings	Aqueous electrolyte concentration (M)				
	Calcium acetate	β-glycerophosphate disodium	Strontium acetate	Cobalt acetate	Sodium fluoride
TiCP	0.05	0.02	-	0.04	0.1
TiCP-S6	0.05	0.02	0.05	0.04	0.1
TiCP-S11	0.05	0.02	0.10	0.04	0.1
TiCP-S18	0.05	0.02	0.15	0.04	0.1

Table S2. Primers used for qRT-PCR.

Gene	Forward primer sequence (5′-3′)	Reverse primer sequence (5′-3′)
Runx2	TGGTGTTGACGCTGATGGAA	ATACCGCTGGACCACTGTTG
BSP	GTCAGAACTGCTGGGACTCG	TGGCATTAGGTGTACTTGACAGT
ALP	CTGAGCGTCCTGTTCTGAGG	GTTCCTGGGTCCCCTTTCTG
OPN	GTGTACCCCACTGAGGATGC	CACGTGTGAGCTGAGGTCTT
OCN	CTTCGTGTCCAAGAGGGAGC	CAGGGGATCCGGGTAAGGA
Col-I	TGCAGGGCTCCAATGATGTT	TGCAGGGCTCCAATGATGTT
HIF-1a	CGATGACACGGAAACTGAAG	CAGAGGCAGGTAATGGAGACA
VEGF	TTGAGTTGGGAGGAGGATGT	TGGCAGGCAAACAGACTTC
GAPDH	ATCAAGTGGGGTGATGCTGG	TACTTCTCGTGGTTCACGCC

Table S3. Elemental composition of the coatings surfaces detected by XPS.

Coatings	Elemental composition (wt.%)						
	Ti	O	Ca	P	Sr	Co	F
TiCP	42.5±1.0	37.1±1.2	6.1±0.5	4.7±0.6	-	5.4±0.7	4.2±0.4
TiCP-S6	38.6±1.1	35.2±1.3	5.8±0.3	4.5±0.5	6.3±0.7	5.5±0.4	4.1±0.3
TiCP-S11	35.8±0.9	33.6±1.0	5.6±0.6	4.6±0.5	11.4±0.6	5.2±0.6	3.8±0.2
TiCP-S18	32.3±1.2	31.4±0.9	5.5±0.5	4.2±0.7	17.6±0.9	5.1±0.6	3.9±0.3

Table S4. Roughness values and contact angles of pristine Ti and the coatings.

Coatings	Roughness (nm)			Contact angle (deg.)	BET surface areas (m^2/g)
	Ra	RMS	Rz		
Ti	7.2±2.1	8.9±1.7	36.8±4.9	125.4±5.1	0.13
TiCP	462.7±41.8	456.9±41.8	1273.4±141.6	47.6±4.1	5.65
TiCP-S6	459.3±42.5	481.3±37.6	1231.2±138.5	46.3±4.5	5.49
TiCP-S11	460.6±39.2	478.6±34.5	1213.6±151.8	48.1±3.9	5.58
TiCP-S18	468.4±40.6	485.7±36.8	1206.8±146.2	45.7±5.2	5.61
